# Phytic Acid in Brown Rice Can Be Reduced by Increasing Soaking Temperature

**DOI:** 10.3390/foods10010023

**Published:** 2020-12-23

**Authors:** Ayaka Fukushima, Gun Uchino, Tatsuki Akabane, Ayaka Aiseki, Ishara Perera, Naoki Hirotsu

**Affiliations:** 1 Graduate School of Life Sciences, Toyo University, 1-1-1 Izumino, Itakura-machi, Oura-gun, Gunma 374-0193, Japan; s39101900193@toyo.jp (A.F.); s39102000016@toyo.jp (T.A.); 2 Faculty of Life Sciences, Toyo University, 1-1-1 Izumino, Itakura-machi, Oura-gun, Gunma 374-0193, Japan; s19101800213@toyo.jp (G.U.); s19101800022@toyo.jp (A.A.); 3 Grain Legume and Oil Crops Research and Development Centre, Department of Agriculture, Angunakolapelessa 82220, Sri Lanka; isharauip@gmail.com

**Keywords:** germination, phytase, phytic acid, rice

## Abstract

Phytic acid (PA) is a storage form of phosphorus in seeds. Phytase enzyme is activated at germination and hydrolyses PA into *myo*-inositol and inorganic phosphate. PA inhibits the absorption of minerals in the human intestine by chelation. Its degradation, therefore, is a key factor to improve mineral bioavailability in rice. Germinated brown rice (GBR) is favoured because it improves the availability of nutrients, and thus have a positive effect on health. In this study, we show the effects of soaking temperature on phytase activity and PA content in GBR. Rice phytase showed thermostability and its activity peaked at 50 °C. After 36 h of soaking, phytase activity was significantly increased at 50 °C and PA content was significantly decreased, compared to that at 30 °C. Zinc (Zn) analysis revealed that there was no significant difference in Zn content among different temperature treatments. Calculated total daily absorbed Zn (TAZ) was significantly higher in GBR compared with non-soaked seeds. Moreover, brown rice grains germinated at 50 °C showed a higher TAZ value than that at 30 °C. Seed germination and seed water soaking at high temperatures reduce PA content in brown rice showing a potentially effective way to improve mineral bioavailability in brown rice.

## 1. Introduction

The world population is steadily increasing and is estimated to reach approximately 10.9 billion by 2100 [1]. To support this rise in population, there is a need to increase the demand for food supply and production. Cereals, for example, are highly sought as a staple crop for maintaining human lives, especially considering that half of the world’s population depends on rice [2]. Brown rice contains phytochemical compounds, including dietary fibre, phenolic acids, flavonoids, tocopherols, minerals, vitamins, amino acid, and γ-oryzanol [3]. These nutritive ingredients help reduce blood pressure, prevent cancer [4,5] and heart disease [6], and have antioxidative effects [7]. Therefore, the consumption of brown rice, instead of white rice, is increasingly becoming the focus of healthy diet recommendations, especially in Asian countries [8].

Phytic acid (*myo*-inositol hexakisphosphate; PA) or IP6 is the major storage form of phosphorus (P) in cereal grains, such as rice, wheat, maize, and barley. It is also considered as a food antinutrient since it chelates micronutrients, including iron, magnesium and zinc (Zn) [9], rendering them impossible to be absorbed by non-ruminants. In fact, PA accounts for 75% of total rice seed-P and is the cause of impaired absorption of micronutrients in human intestines [10]. Lönnerdal et al. [11] showed that a diet high in PA negatively affects Zn absorption and suggested that lower PA content in cereal grains would contribute to enhanced Zn availability for absorption by humans.

PA is digested by phytase to generate inorganic phosphate (Pi) and lower *myo*-inositol phosphate (IP2, IP3, IP4, and IP5). It is known that phytase is induced during germination to convert PA in rice grains to Pi, which is utilized during seed germination [12]. To improve mineral bioavailability, rice with lower PA content is of increasing interest [13]. PA is mainly accumulated in the aleurone layer of cereal grains [14]. Therefore, reducing PA content without the need for polishing out the aleurone layer is an efficient way to improve mineral bioavailability in rice. Although, the genetic and environmental factors controlling P content in rice have been investigated, including P fertilizers, there is still not enough information to help understand the dynamics of P contents in brown rice [15,16]. Low phytic acid (*lpa*) mutant rice has been developed to increase mineral bioavailability [17,18,19]. However, *lpa* rice has been reported to have lower germination rates and seedling vigor, and further studies are needed to solve this problem.

Germinated brown rice (GBR) which contains the functional nutrients is considered as excellent nutritional food. GBR is favoured because it not only promotes beneficial biological activities for better health but also is superior to cook because of better water absorption and good chewiness compared to non-germinated brown rice [8,20]. Moreover, germination of rice grains might change various nutrient contents, such as sugar, vitamin B, total protein, and γ-aminobutyric acid (GABA) [21]. At the germination phase, phytase is induced inside the seed and PA is degraded [22,23]. The changes in phytase activity have been analysed in several cereals, showing that PA content decreases during germination [24,25]. Considering this, phytase might have important potential to improve the mineral bioavailability of GBR by reducing PA content. However, little information is reported for the production of GBR with low PA content.

Phytase also plays an important role in the livestock feed industry. Application of phytase (Ronozyme^®^ P5000 CT) derived from *Peniophora lycii* is effective to prevent eutrophication [26]. Phytases are found in natural sources including not only plants but also microbes. In fact, microbial phytase retains its activity when exposed to high temperatures compared with plant phytase [27]. Parhanfar et al. [28] showed that phytase from *Bacillus* sp. DM12 retained its activity at a temperature range of 30–80 °C. Thus, thermostable phytase identified from microbial sources is applicable in food and animal feed industries.

Phytase from plants is not applied in seed processing to produce low PA GBR because its thermostability is still unclear. In this study, we investigated the effect of different temperatures at soaking on PA content and phytase activity. Zn availability was also estimated.

## 2. Materials and Methods

### 2.1. Seed Germination

We used Japonica brown rice cultivar “Koshihikari” grown in paddy fields at Itakura, Gunma, Japan in 2018. The dehusked brown rice was sterilized by sodium hypochlorite. Then, ca. 120 grains in each temperature treatment were soaked in plastic Petri dishes filled with deionized water and incubated for 10 days. To compare the effect of temperature on soaking, grains were soaked at 30 °C, 35 °C, 40 °C, 45 °C, 50 °C, and 55 °C in an incubator (MIR-554, SANYO Electric Co., Ltd., Osaka, Japan). Deionized water was renewed every two days. We collected grains at 2, 4, 6, 8, and 10 days and stored them at −80 °C for the determination of PA content and phytase activity (*n* = 3–5). For short-time analysis of PA content and phytase activity, ca. 50 grains were soaked at 30 °C and 50 °C and collected every 12 h up to 48 h and stored at −80 °C for analysis.

### 2.2. Phytase Activity Assay

Phytase activity was determined based on Azeka et al. [24] and Ou et al. [29] with modification. Unless stated otherwise, all chemicals were obtained from Nacalai tesque (Kyoto, Japan). One grain was homogenized with 1 mL of 0.5 M sodium acetate buffer, then centrifuged at 15,300× *g* for 10 min at 4 °C. The supernatant was collected for enzyme activity assay. Sodium acetate buffer and 0.1 g/L sodium phytate (Sigma-Aldrich, St. Louis, MO, USA) were added into a 1.5 μL tube and mixed. The enzyme reaction was initiated by adding crude enzyme into the mixed buffer solution. After incubation, enzyme reaction was terminated with trichloroacetic acid (50% *w/v*) and centrifuged using high speed refrigerated micro centrifuge (MX-305, Tomy, Tokyo, Japan) at 15,300× *g* for 10 min at 4 °C. Enzymatic reaction was measured at 50 °C and at soaking temperature of the sample as above. Pi concentration was measured at 655 nm after adding a colour reagent containing ascorbic acid (10% *w/v*), concentrated H_2_SO_4_, and ammonium molybdate (5% *w/v*). Mixed buffer solution without crude enzyme was used as a blank. To determine the Pi concentration, calibration curves were prepared with 0.03 M KH_2_PO_4_. Enzyme activity (U) was expressed as 1 μmol of Pi released from IP6 per minute. Total protein contents were measured using the same extract following the Bradford method [30].

### 2.3. Determination of PA

PA content was measured as reported in a previous study [31]. Phytase digested PA into lower *myo*-inositol phosphate forms and Pi. Final Pi is released by treatment of alkaline phosphatase. We measured Pi released from PA and calculated the PA content following the manufacturer’s guidelines in the phytic acid (phytase)/total phosphorus assay kit (K-PHYT, Megazyme International, Wicklow, Ireland). One germinated seed was crushed with 0.66 M HCl and then neutralized with 0.75 M NaOH. Assay solution and extracted PA with and without enzyme were incubated at 40 °C for 1 h. Colour reagent for detecting Pi as described above was added prior to recording absorbance at 655 nm.

### 2.4. Determination of Zn Content

Zn content was measured by inductively coupled plasma mass spectrometry (ICPMS, XSERIES 2, Thermo Fisher Scientific, Waltham, MA, USA) as previously reported [31]. One rice grain was digested with 4 mL of HNO_3_ between 80 °C and 150 °C and filtered by syringe. Diluted digested samples were analysed according to the manufacturer’s protocol.

### 2.5. Data Analysis

The data were analysed by JMP software (SAS Institute, Cary, NC, USA). Tukey’s HSD test was used for the determination of significant differences between treatment means at *p* < 0.05.

## 3. Results

### 3.1. The Effect of Temperature on Soaking

**Figure 1:**
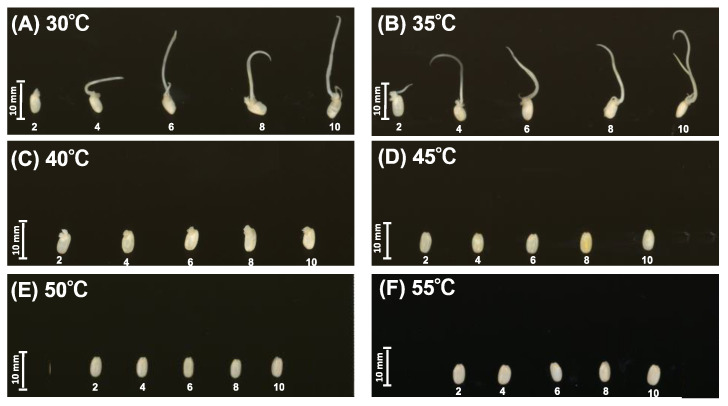
Effect of temperature on germination. (**A**) 30 °C, (**B**) 35 °C, (**C**) 40 °C, (**D**) 45 °C, (**E**) 50 °C, and (**F**) 55 °C.

Germination was strongly affected by temperature (Figure 1). At 30 °C and 35 °C, the coleoptile and root emerged 2 days after soaking (Figure 1A,B). No germination was observed at 45 °C, 50 °C, and 55 °C (Figure 1D–F). At 40 °C, small buds appeared 2 days after soaking but the germ did not elongate until 10 days after soaking (Figure 1C).

### 3.2. Optimum Temperature of Phytase Activity Derived from Non-Soaked Grain

**Figure 2:**
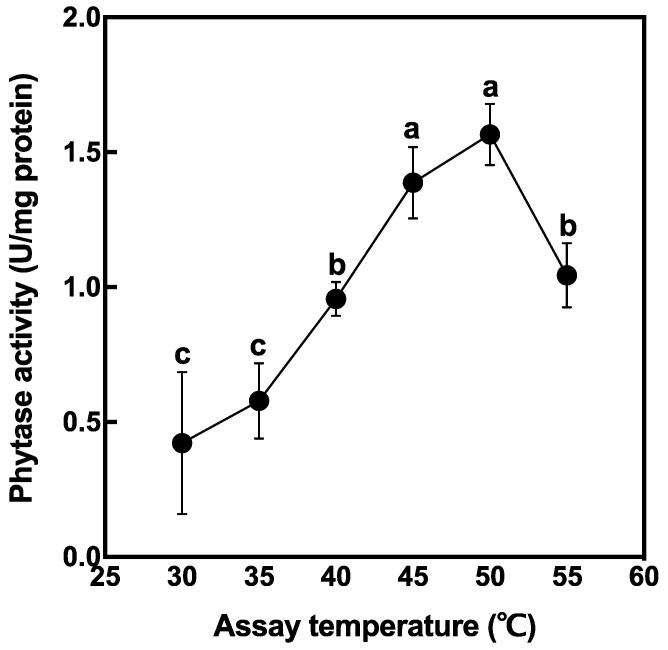
Phytase activity extracted from brown rice. Data are shown as the mean ± standard deviation from three replicates. Different letters indicate statistical differences (Tukey’s HSD test, *p* < 0.05) between treatments.

The crude enzyme was extracted from non-soaked brown rice and phytase activity was measured at different temperatures (Figure 2). Phytase activity in rice grain tended to increase with temperature elevation and peaked at 50 °C. There was no significant difference in phytase activity between 45 °C and 50 °C. From this result, further enzyme reactions were conducted at 50 °C for the evaluation of maximum phytase activity.

### 3.3. The Effect of Soaking Temperature on Phytase Activity and PA Content

**Figure 3:**
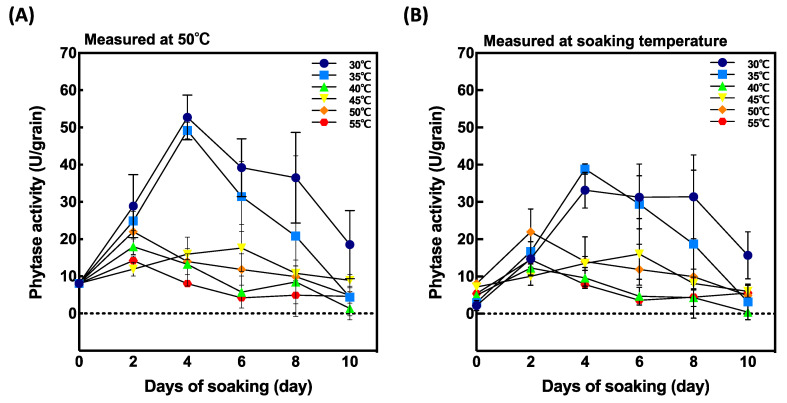
Effect of soaking temperature on phytase activity in germinated brown rice (GBR). (**A**) phytase activity measured at 50 °C and (**B**) phytase activity measured at soaking temperature. Data are shown as the mean ± standard deviation from 3–5 replicates.

Phytase activity was measured at 50 °C and at the soaking temperature (Figure 3). When measured the maximal activity at 50 °C (Figure 3A), the samples soaked at 30 °C and 35 °C for 4 days after soaking showed highest activities. Although phytase activity initially increased with soaking temperature, it gradually decreased with increasing temperature from 45 °C to 55 °C.

To evaluate the effect of soaking temperature on phytase activity, we measured phytase activity at soaking temperature (Figure 3B). Although a similar tendency was observed as when measured at 50 °C, but they were lower than that measured at 50 °C.

**Figure 4:**
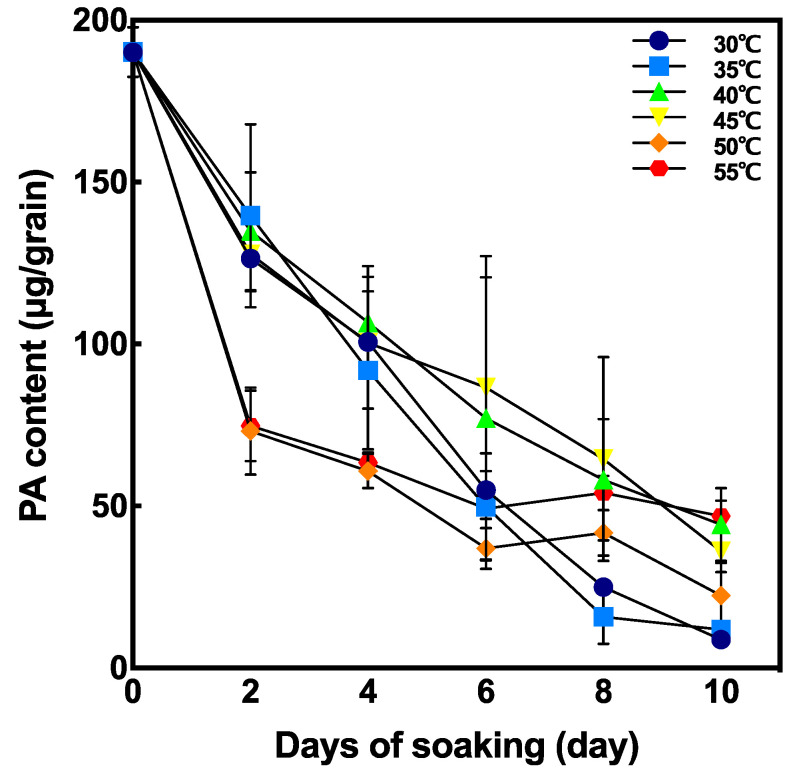
Effect of soaking temperature on phytic acid (PA) content in germinated brown rice (GBR). Data are shown as the mean ± standard deviation of 3–5 replicates.

The changes in PA content in brown rice during soaking is shown in Figure 4. PA content after soaking at 50 °C and 55 °C were significantly lower at 2 days after soaking, whereas the degradation of PA content in grains soaked at 30 °C, 35 °C, 40 °C, and 45 °C was slower than that at 50 °C and at 55 °C.

### 3.4. The Effect of Soaking Temperature on Phytase Activity and PA Content within 48 h

**Figure 5:**
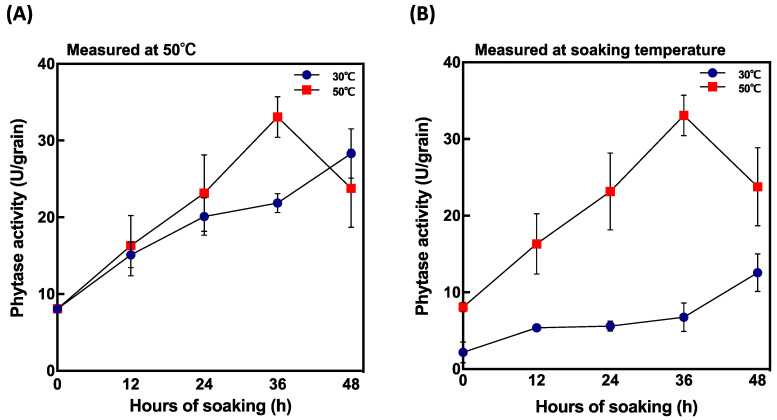
Changes in phytase activity in short periods after soaking at 30 °C and 50 °C. (**A**) phytase activity measured at 50 °C and (**B**) phytase activity measured at soaking temperature. Data are shown as the mean ± standard deviation from 3–5 replicates.

Phytase activity was highest 4 days after soaking at 30 °C and 35 °C (Figure 3A). To determine the difference in the peak of activity between soaking temperature, phytase activity and PA content were measured in the grains within 48 h after soaking at two different soaking temperatures, 30 °C and 50 °C. The highest enzyme activity was recorded 36 h after soaking when grains were soaked at 50 °C (Figure 5A) and was significantly different from that recorded at 30 °C. Comparing phytase activity and soaking temperature (Figure 5B), grains soaked at 50 °C showed higher phytase activity than those soaked at 30 °C. Furthermore, phytase was induced faster at 50 °C after 36 h soaking compared to at 30 °C.

**Figure 6:**
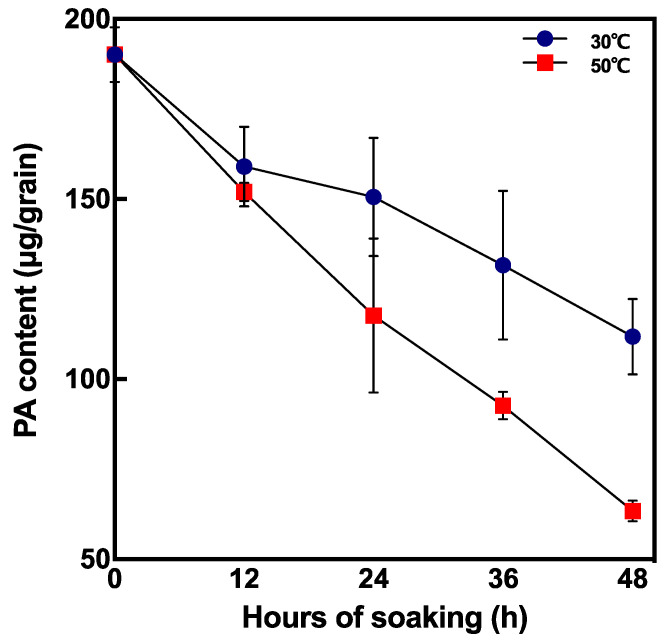
Changes in PA content in short periods after soaking at 30 °C and 50 °C. Data are shown as the mean ± standard deviation from 3–5 replicates.

PA content during soaking drastically decreased at 50 °C 48 h after soaking compared with non-soaked brown rice (Figure 6). To reduce half of the original PA content, it took 96 h and 24–36 h by incubating at 30 °C and 50 °C, respectively.

### 3.5. Estimation of Zn Bioavailability in GBR

Zn and PA contents and calculated TAZ [31] are shown in Table 1. There were no significant differences in Zn content between non-soaked brown rice, grains soaked at 30 °C, and those soaked at 50 °C (Table 1). TAZ in grains soaked at 50 °C was significantly higher than that in non-soaked brown rice or grains germinated at 30 °C.

## 4. Discussion

### 4.1. The Thermal Stability of Phytase

Few investigations have been reported involving optimum temperatures for intrinsic phytase derived from rice grain [32]. In this study, we investigated the optimum temperatures for phytase induction in rice grains. Our results showed that phytase activity was temperature-dependent and peaked at 50 °C (Figure 2). Generally, heat stability of phytase was observed at 50–100 °C in phytase derived from diverse microbial sources [28,33,34,35,36,37,38]. The activity of commercially derived microbial phytase from *Aspergillus niger* and *Saccharomyces cerevisiae* was the highest at 45 °C and between 50 °C and 60 °C, respectively [39]. These results suggested that the thermostability of phytase from the rice was similar to that of microorganisms.

It was observed that phytase did not reach its maximal potential at ambient germination temperature. Our results showed that phytase activity at 30 °C decreased to roughly 40% compared with that at the optimum temperature (Figure 3B). On the other hand, phytase activity at 30 °C and 35 °C was significantly higher than that at other higher temperatures after 4 days of soaking (Figure 3A). Phytase activity was reduced above 40 °C, below the optimal temperature of phytase itself. Considering that the natural germination process takes place at ambient temperature, 30 °C might be identified as the optimal temperature for phytase activity in germinating rice grains. Phytase activity might have been reduced by less thermostability above 40 °C, leading to no germination.

### 4.2. Effect of Temperature on PA Degradation

Similar to our results (Figure 4), germinated seeds and seedlings have been shown to have low PA content in cereals including rye and barley [25,40], soybean [41], sorghum [42], rice [43], and several crops [24]. In general, brown rice is incubated for 1–2 days at ambient temperature to produce GBR [44], PA content is reduced to only about 30% for 2 days after soaking. However, this stage of GBR has elongated coleoptile and is not suitable for consumption.

In our study, although no germination was observed at 50 °C and 55 °C, PA content greatly decreased at 2 days after soaking. This result might suggest that at higher temperatures the peak of PA degradation is reached much earlier. To investigate PA degradation in shorter periods, PA content and phytase activity were compared at 30 °C and 50 °C at 12, 24, 36, and 48 h after soaking (Figure 5 and Figure 6). Phytase activity was significantly increased at 50 °C and 36 h after soaking compared to that at 30 °C (Figure 5B). Moreover, PA content decreased by 70.3% at 50 °C 48 h after soaking (Figure 6). According to our results, seed soaking at high temperatures (>50 °C) enables rapid PA degradation within 48 h of soaking.

### 4.3. To Produce High Zn Bioavailable GBR

In this study, we observed that phytase activity and PA degradation peaked relatively earlier at high temperatures (50 °C) and that high temperatures inhibit seed germination. PA content in brown rice grains soaked at 30 °C and 35 °C, and 6 days after soaking, was almost similar to that in grains incubated at high temperatures (50 °C and 55 °C) and 2 days of soaking; however, a 6-day germination is not suitable because they have long coleoptile as GBR. On the other hand, small germ development in GBR at lower temperatures (e.g., 2 days after soaking at 30 °C) did not reduce PA content and mineral bioavailability could be considered as not improved. At high temperatures, PA drastically decreased within 36 h, but germ and root developments were not observed. Thus, brown rice incubated at high temperatures can reduce PA content without sprouting.

Our previous study showed that low PA rice had high Zn bioavailability rather than high PA rice among the natural variation of rice [31]. According to the results, low PA traits rather than high Zn content would be key for the production of low PA rice. To reduce PA content in rice, milling is the most frequently used technique. However, since beneficial nutrients in brown rice are also reduced by milling [45], it is desirable to reduce PA content without milling to improve mineral bioavailability.

TAZ value is a key indicator to estimate Zn bioavailability. In brown rice, low PA rice has a high TAZ value. Based on this, we hypothesized that GBR treated at high temperatures might improve TAZ. To test the hypothesis, we analysed Zn content in grains incubated for 36 h at 30 °C and 50 °C and calculated TAZ (Table 1). There were no significant differences in Zn contents between the treatments, showing that these soaking conditions did not significantly affect Zn content. Although PA content was reduced after soaking, especially at 50 °C, TAZ increased 2.25 times at 50 °C for 36 h soaking. This result shows that PA content can be decreased without seed germination, and that TAZ can be improved by simple thermal treatment. Although the soaking time and temperature to be tuned by rice varieties, soaked brown rice treated at 50 °C for 36 h can be considered to improve mineral bioavailability by reducing PA without losing beneficial nutrients. Practically, this cost-effective and generic procedure has the potential in helping to solve the global nutritional problems.

## 5. Conclusions

Phytase activity in soaked grain showed thermostability. Different incubation temperatures have varied influences on seed germination and PA degradation. Significant degradation of PA was observed at higher (>50 °C) incubation temperatures. PA content was significantly decreased at 50 °C, while Zn content did not change with soaking temperature. Our study showed that water soaking at 50 °C improved TAZ. To improve Zn bioavailability in rice, germination and seed imbibition is a possible way to reduce PA content without losing minerals in bran during rice milling.

## Figures and Tables

**Table 1:** Zn bioavailability of non-soaked and soaked grains at 30 °C and 50 °C 36 h after soaking. Data are shown as the mean ± standard deviation from three replicates. Values in parentheses indicate the percentage of the mean value in non-soaked grains. Different letters indicate statistical differences (Tukey’s HSD test, *p* < 0.05) between treatments.

Treatment	PA Content (μg/g)		Zn Content (µg/g)		TAZ (mg/d)	
Non-soaked grain	190.2 ± 7.6 ^a^	(100)	39.6 ± 3.5 ^a^	(100)	1.4 ± 0.1 ^c^	(100)
Soaked grain (30 °C, 36 h)	147.8 ± 20.9 ^b^	(69)	42.3 ± 2.5 ^a^	(107)	2.2 ± 0.2 ^b^	(156)
Soaked grain (50 °C, 36 h)	99.0 ± 17.0 ^c^	(49)	39.6 ± 3.2 ^a^	(100)	3.1 ± 0.2 ^a^	(225)

## Data Availability

The data that support the findings of this study are available from the corresponding author upon reasonable request.
